# A prospective study of remote delirium screening using the modified K-4AT for COVID-19 inpatients

**DOI:** 10.3389/fpsyt.2022.976228

**Published:** 2022-08-18

**Authors:** Hyun Jung Hur, Yu Na Jang, Hye Yoon Park, Young Seok Lee, Du Hyun Ro, Beodeul Kang, Kyoung-Ho Song, Hye Youn Park

**Affiliations:** ^1^Department of Neuropsychiatry, Seoul National University Bundang Hospital, Seoul, South Korea; ^2^Department of Psychiatry, Seoul National University Hospital, Seoul National University College of Medicine, Seoul, South Korea; ^3^Division of Pulmonary, Allergy, and Critical Care Medicine, Department of Internal Medicine, Korea University Guro Hospital, Seoul, South Korea; ^4^Department of Orthopedic Surgery, Seoul National University Hospital, Seoul, South Korea; ^5^Medical Oncology, Department of Internal Medicine, CHA Bundang Medical Center, CHA University School of Medicine, Seongnam, South Korea; ^6^Division of Infectious Diseases, Department of Internal Medicine, Seoul National University Bundang Hospital, Seoul National University College of Medicine, Seongnam, South Korea

**Keywords:** COVID-19, delirium, 4AT, remote screening, delirium assessment tools

## Abstract

**Background:**

Delirium is a neuropsychiatric condition strongly associated with poor clinical outcomes such as high mortality and long hospitalization. In the patients with Coronavirus disease 2019 (COVID-19), delirium is common and it is considered as one of the risk factors for mortality. For those admitted to negative-pressure isolation units, a reliable, validated and contact-free delirium screening tool is required.

**Materials and methods:**

We prospectively recruited eligible patients from multiple medical centers in South Korea. Delirium was evaluated using the Confusion Assessment Method (CAM) and 4‘A’s Test (4AT). The attentional component of the 4AT was modified such that respondents are required to count days, rather than months, backward in Korean. Blinded medical staff evaluated all patients and determined whether their symptoms met the delirium criteria of the Diagnostic and Statistical Manual of Mental Disorders 5 (DSM-5). An independent population of COVID-19 patients was used to validate the 4AT as a remote delirium screening tool. We calculated the area under the receiver operating characteristic curve (AUC).

**Results:**

Out of 286 general inpatients, 28 (9.8%) inpatients had delirium. In this population, the patients with delirium were significantly older (*p* = 0.018) than the patients without delirium, and higher proportion of males were included in the delirium group (*p* < 0.001). The AUC of the 4AT was 0.992 [95% confidence interval (CI) 0.983–1.000] and the optimal cutoff was at 3. Of the independent COVID-19 patients, 13 of 108 (12.0%) had delirium. Demographically, the COVID-19 patients who had delirium only differed in employment status (*p* = 0.047) from the COVID-19 patients who did not have delirium. The AUC for remote screening using the 4AT was 0.996 (0.989–1.000). The optimal cutoff of this population was also at 3.

**Conclusion:**

The modified K-4AT had acceptable reliability and validity when used to screen inpatients for delirium. More importantly, the 4AT efficiently screened for delirium during remote evaluations of COVID-19 patients, and the optimal cutoff was 3. The protocol presented herein can be used for remote screening of delirium using the 4AT.

## Introduction

During the Coronavirus disease 2019 (COVID-19) pandemic, the prevalence of acute mental change or delirium in COVID-19 patients ranged from 10% to 70% (1–4). Delirium, defined as fluctuating cognitive disturbance, in critically ill patients is strongly associated with poor outcomes, and a recent meta-analysis confirmed that delirium in COVID-19 patients was significantly associated with high mortality (5). As delirium is associated with long-term hospitalization, high medical costs, and high mortality (6), close monitoring of COVID-19 patients for early delirium detection is crucial. Although the prevalence of delirium in COVID-19 patients has been reported in many studies (1–4), some of which highlighted the importance of delirium screening (5), few studies have specifically explored how to assess features of delirium. The limited patient contact, shortage of trained healthcare professionals, and restrictions on family visits were speculated as barriers to routine assessment and early detection of delirium (7). Thus, a detailed method for delirium screening in COVID-19 patients is essential; any such method must consider COVID-19 patient-specific circumstances, such as isolation in negative pressure units.

The 4 ‘A’s test (4AT) is a delirium screening tool (8). Assessment using the 4AT is brief and simple; clinical experience is not required. In contrast, the Confusion Assessment Method (CAM), which has been widely used and validated in many languages (9), is relatively complex and must be administered by a skilled professional. Moreover, as 4AT delirium screening does not require physical contact with the patient, using 4AT as a delirium assessment tool for COVID-19 patients could be useful. However, the 4AT has not yet been validated for any patient groups in South Korea, and the attention component requires modification during the translation from the English to Korean version (K-4AT), because the two task versions differ in terms of difficulty. Thus, in this study, we first validated the K-4AT in general inpatients. Then, we focused on COVID-19 inpatients who required remote screening. Finally, we present a contact-free delirium screening protocol for COVID-19 patients.

## Materials and methods

### Subjects

**FIGURE 1:**
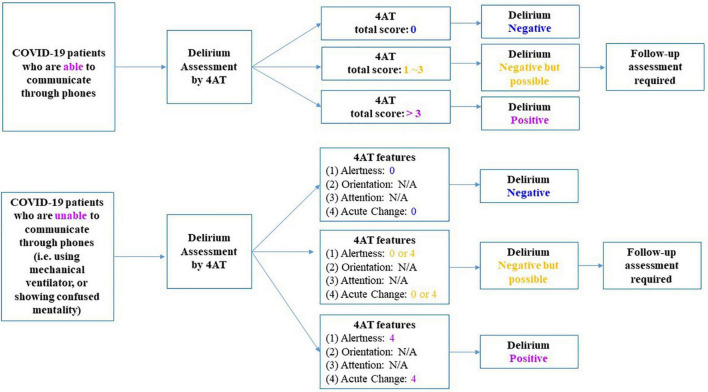
Protocol of delirium screening by 4‘A’s Test (4AT) for Coronavirus disease 2019 (COVID-19) inpatients. 4AT, 4‘A’s Test; COVID-19, Coronavirus disease 2019.

In order to evaluate the reliability and validity of the modified K-4AT, adult general inpatients were recruited from the intensive care unit, postoperative unit or progressive cancer unit of seven South Korean medical centers. The recruitment period was from March to December 2021. Patients who were unable to communicate verbally, or who were diagnosed with dementia or cognitive impairment, were excluded. Patients were recruited only when informed consent could be obtained from them or their family members. The Institutional Review Board of each center approved this part of the study. Patients with COVID-19 diagnoses were independently recruited on admission to the Seoul National University Bundang Hospital or Seoul National University Hospital for treatment of COVID-19-related symptoms. Recruitment and screening proceeded via two steps. First, as all patients were isolated in COVID-19 units, verbal consents were obtained via interphones or their cellphones after being informed about the study. Delirium assessments were then performed. The detailed flowchart of remote delirium assessment by 4AT was elaborated in Figure 1. In the next step, patients who had previously given verbal consent were discharged and thus able to meet researchers face-to-face. We obtained written consent at this time. The protocol was approved by the Institutional Review Boards of both centers (joint approval no. 2103-675-302).

### Assessments

Researchers assessed the clinical features of all recruited general inpatients and COVID-19 patients to determine whether they were eligible for the study. For eligible patients who gave informed consent, age, sex, educational level, marital status, comorbidities including the current diagnosis, and diagnostic history data were recorded. Two independent researchers of each medical center evaluated delirium using the CAM and 4AT, respectively (10, 11). Before the commencement of this study, in order for all researchers and health care professionals to administer CAM, multiple workshops and training sessions for delirium evaluation by CAM and CAM-ICU were held. In workshops and sessions, board certified psychiatrists shared case examples and studied actual evaluations through videotaped records. The CAM is a validated and widely used tool; many versions are available. We employed the CAM-ICU, which includes the Richmond Agitation and Sedation Scale (RASS) and also assesses acute onset, inattention, fluctuating cognition, and disorganized thinking (12). The final decision by CAM is either delirium positive or delirium negative. When acute onset and inattention features are positive and either fluctuating cognition or disorganized thinking is positive, the patient is determined as delirium present. Although the 4AT has similar features with CAM, the 4AT is designed as scoring system. The 4AT includes four features. The first feature evaluates alertness by observation. The second and the third features assess cognition and attention via brief cognitive tasks, which are modules of the Abbreviated Mental Test 4 (AMT4) (8). The last feature evaluate the acute change or fluctuating course of mental status within last 24 h. The total score is calculated by adding up scores of each feature and the score range is minimum 0 to maximum 12. The original English version of 4AT has cutoff at 4, meaning that when the total score is greater than 3, the patient is considered as delirium present. To ensure that the English and Korean versions of the tasks were equally challenging, the attention component was modified such that the participants had to count days backwards, rather than months, as counting months in Korean is the same as reciting numbers from 12 to 1. Apart from these assessments, medical staffs including doctors and nurses blindly evaluated features of delirium on the assessment days. These bedside evaluations involved patient contact and were based on the Diagnostic and Statistical Manual of Mental Disorders 5 (DSM-5) delirium criteria (13). In order to make the diagnosis of delirium based on DSM-5, inpatient physician, nurses, and consultation-liaison psychiatrists of each center obtained information from family members regarding patients’ mental change or fluctuation and also reviewed medical charts. Additionally, the direct cognitive tasks from the Korean version of Mini-Mental State Exam are asked to the patients, such as registration and recall of words, orientation and serial sevens (14).

### Statistical analysis

We compared the demographic data of the patients with and without delirium using the Pearson χ2 test and Fisher’s exact test for categorical variables, and the t-test for continuous variables. We calculated the Cronbach’s alpha as a measure of reliability of the modified K-4AT, and generated receiver operating characteristic (ROC) curves to analyze validity. In this analysis, the DSM-5 based diagnoses were set as a true value. Sensitivity, specificity, and accuracy were calculated at various cutoffs; the optimal cutoff could be determined at the point of maximized sensitivity and specificity. For the COVID-19 population, the same analyses were performed to determine the reliability and validity of remote 4AT assessment, and the effectiveness of CAM and 4AT. We used R (pROC ver. 1.17.0; Microsoft R Open Version 3.6.2. Microsoft and R Core Team, Microsoft Corporation, Redmont, WA, United States) for the analyses; *p*-values < 0.05 were considered significant. Interrater reliability was determined according to the extent of the agreement in delirium diagnoses based on DSM-5 criteria between the two raters. Cohen’s kappa showed that the agreement was substantially consistent [κ = 0.710; 95% confidence interval (CI) = 0.412–1.000; *p* < 0.001].

## Results

### Hospitalized general patients and contact delirium screening

Of 286 general inpatients, 28 (9.8%) had delirium diagnosis based on DSM-5 and 258 (90.2%) did not. Demographically, the two groups differed significantly in age and gender only (Table 1). Patients with delirium were significantly older (mean age = 73.21 years, standard deviation = 10.56 years) than those without delirium (*p* = 0.018), and there was a higher proportion of males in the delirium group (*p* < 0.001). There were significant group differences according to diagnoses (*p* < 0.001). The Cronbach α of the 4AT was 0.786, indicating high internal reliability. The optimal cutoff for the K-4AT was 3, at which the sensitivity was 1.000 (95% CI = 1.000-1.000), the specificity was 0.980 (95% CI = 0.960–0.996), and the accuracy was 0.982 (95% CI = 0.964–0.996) (Table 2). The area under the ROC curve (AUC) was 0.992 (95% CI = 0.983–1.000). On the other hand, the sensitivity of CAM was 0.643 (95% CI = 0.464-0.821), the specificity was 0.981 (95% CI = 0.961-0.996), and the accuracy was 0.948 (95% CI = 0.923–0.969). The AUC was 0.812 (95% CI = 0.721–0.903), which was lower than AUC of 4AT.

**TABLE 1:** Demographics between delirious and non-delirious patients diagnosed by the Diagnostic and Statistical Manual of Mental Disorders 5 (DSM-5) in general inpatient population.

Variables	Delirium (*n* = 28)	Non-delirium (*n* = 258)	*p*
Age (years)^a^	73.21 ± 10.56	67.51 ± 12.17	0.018*
Sex, Female^b^	9 (32.1%)	186 (72.1%)	<0.001***
Employment status, employed^b^	6 (21.4%)	63 (24.5%)	0.717
Educational level^b^			0.801
No school	1 (4.2%)	7 (2.8%)	
Primary	4 (16.7%)	64 (25.5%)	
Secondary	5 (20.8%)	50 (19.9%)	
High level	8 (33.3%)	75 (29.9%)	
Degree	6 (25.0%)	55 (21.9%)	
Marital status, married^b^	28 (100.0%)	234 (95.9%)	0.606
Primary diagnosis			<0.001***
Intensive care unit	15 (53.6%)	65 (25.2%)	
Post-operative unit	2 (7.1%)	142 (55.0%)	
Progressive cancer unit	11 (39.3%)	51 (19.8%)	

^a^Data given as mean ± standard deviation. ^b^Data given as number (%).

*p < 0.05, *** p < 0.001.

**TABLE 2:** Sensitivity, specificity, and accuracy of the 4‘A’s Test (4AT) and the Confusion Assessment Methods (CAM) in general inpatient population (*n* = 286).

4AT cutoff score	Sensitivity (95% CI)	Specificity (95% CI)	Accuracy
0	1.000 (1.000, 1.000)	0.000 (0.000, 0.000)	0.094 (0.094, 0.094)
1	1.000 (1.000, 1.000)	0.888 (0.848, 0.928	0.899 (0.862, 0.935)
2	1.000 (1.000, 1.000)	0.960 (0.932, 0.984)	0.964 (0.938, 0.986)
**3**	**1.000 (1.000, 1.000)**	**0.980 (0.960, 0.996)**	**0.982 (0.964, 0.996)**
4	0.962 (0.885, 1.000)	0.988 (0.972, 1.000)	0.986 (0.971, 0.996)
5	0.731 (0.539, 0.885)	0.988 (0.972, 1.000)	0.964 (0.942, 0.982)
6	0.731 (0.539, 0.885)	0.988 (0.972, 1.000)	0.964 (0.942, 0.982)
7	0.577 (0.385, 0.769)	0.988 (0.972, 1.000)	0.949 (0.928, 0.971)
8	0.346 (0.154, 0.539)	0.992 (0.980, 1.000)	0.931 (0.909, 0.953)
9	0.192 (0.038, 0.346)	1.000 (1.000, 1.000)	0.924 (0.913, 0.949)
10	0.192 (0.038, 0.346)	1.000 (1.000, 1.000)	0.924 (0.909, 0.938)
11	0.192 (0.038, 0.346)	1.000 (1.000, 1.000)	0.924 (0.909, 0.938)
12	0.192 (0.038, 0.346)	1.000 (1.000, 1.000)	0.924 (0.909, 0.938)
CAM-ICU	0.643 (0.464, 0.821)	0.981 (0.961, 0.996)	0.948 (0.923, 0.969)

The best cutoff score (3) appears in bold.

Cronbach’s α coefficient of 4AT = 0.786.

AUC of 4AT = 0.992 (95% CI = 0.983-1.000).

AUC of CAM = 0.812 (95% CI = 0.721-0.903).

### Hospitalized COVID-19 patients and remote delirium screening

**FIGURE 2:**
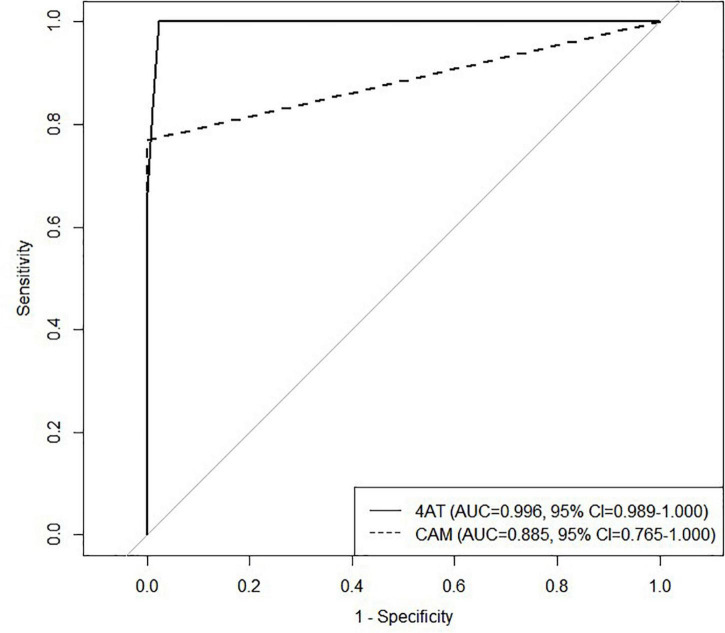
Receiver operating characteristics (ROC) analysis was performed to compare the 4‘A’s Test (4AT) with the Confusion Assessment Methods (CAM) in Coronavirus disease 2019 (COVID-19) inpatients. ROC, Receiver operating characteristics; 4AT, 4‘A’s Test; CAM, Confusion Assessment Methods; COVID-19, Coronavirus disease 2019.

Out of 108 COVID-19 inpatients, 13 (12.0%) had delirium diagnosis based on DSM-5 and 95 (88.0%) did not. Demographically, the two groups differed in employment status only (*p* = 0.047) and there were no significant differences in age, gender, education level, and marital status (Table 3). The Cronbach’s α of the remotely conducted K-4AT was 0.810, indicating high internal consistency. The AUC of remote K-4AT was 0.996 (95% CI = 0.989–1.000) (Figure 2). The optimal cutoff of remote assessment by K-4AT was 3. At this point, the sensitivity was 1.000 (95% CI = 1.000–1.000), the specificity was 0.978 (95% CI = 0.944–1.000), and the accuracy was 0.980 (95% CI = 0.951–1.000). The AUC of the remote CAM was 0.885 (95% CI = 0.765-1.000); the sensitivity was 0.769 (95% CI = 0.539–1.000), the specificity was 1.000 (95% CI = 1.000–1000), and the accuracy was 0.972 (95% CI = 0.944–1000). Thus, for COVID-19 inpatients, remote delirium screening by K-4AT yielded more reliable results than by CAM.

**TABLE 3:** Demographics between delirious and non-delirious patients diagnosed by the Diagnostic and Statistical Manual of Mental Disorders 5 (DSM-5) in Coronavirus disease 2019 (COVID-19) inpatients.

Variables	Delirium (*n* = 13)	Non-delirium (*n* = 95)	*p*
Age (years) ^a^	62.62 ± 10.60	59.04 ± 15.09	0.411
Sex, Female ^b^	4 (30.8%)	41 (43.2%)	0.395
Employment status, employed^b^	11 (84.6%)	45 (55.6%)	0.047*
Educational level^b^			0.525
No school	2 (15.4%)	13 (17.6%)	
Primary	0 (0.0%)	0 (0.0%)	
Secondary	2 (15.4%)	5 (6.8%)	
High level	6 (46.2%)	28 (37.8%)	
Degree	3 (23.1%)	28 (37.8%)	
Marital status, married^b^	11 (84.6%)	80 (84.2%)	0.970

^a^Data given as mean ± standard deviation. ^b^Data given as number (%).

*p < 0.05.

## Discussion

As the 4AT is a brief assessment (< than 3 min) that can be administered by non-professionals (8), we used the K-4AT as an adequate delirium screening tool for a prospective multi-center study. Our study findings showed that the K-4AT proved to be a valid screening tool. Among the general inpatients, 9.8% had delirium. In agreement with earlier studies (15, 16), we found significant differences in age, sex, and diagnoses between the delirious and non-delirious patients. By including patients with three different principal diagnoses, we confirmed the validity and generality of delirium screening by K-4AT. The original 4AT has a cutoff of 4; however, we found that the optimal cutoff was 3. Similarly, a recent study of the Spanish version of the 4AT indicated that the maximized combination of sensitivity and specificity was at a cutoff of 3 (17). These results implied that the modified and translated version of 4AT may be better at detecting delirium when it has strict cutoff.

Of the COVID-19 inpatients admitted to negative pressure isolation units, 12% developed delirium; this rate is similar to those of previous studies (1–4), which can imply that delirium is not uncommon in COVID-19 patients. The recent international cohort study reported that more than half of the COVID-19 patients from the cohort had delirium and more than 80% of them underwent comma status during hospitalization (18). As these COVID-19 patients with delirium are exposed to risk of poor clinical outcomes (5, 6), early detection and prevention is critical. A recent meta-analysis revealed that symptoms of delirium, especially confusion and agitation, were common among patients in the acute stage of severe acute respiratory syndrome (SARS), Middle East respiratory syndrome (MERS), and COVID-19 (19). When dealing with an infectious disease outbreak, control of the spread is crucial but difficult, depending on whether isolation or quarantine be mandated and how long the asymptomatic period is (20). Isolation and quarantine that are sudden environmental changes for patients may be risk factors for delirium onset. An observational study from the early COVID-19 era reported that the mortality of COVID-19 patients with delirium was 10% higher than that of COVID-19 patients without delirium (21); another study reported that COVID-19 patients with delirium were at great risk of 30-day mortality and readmission after discharge (22). Thus, to avoid exacerbation of the clinical symptoms of infectious diseases, early detection and treatment of delirium are imperative. This implied that delirium screening should be routine in patients with respiratory infectious diseases. However, for isolated patients with highly contagious respiratory infections (SARS, MERS, and COVID-19), delirium evaluation on a routine basis is difficult. Studies regarding delirium screening for these patients are still limited.

Thus, we used the K-4AT and remotely assessed COVID-19 inpatients; as with the face-to-face setting, the test was valid and useful, and the cutoff score was 3. In the course of delirium assessment, we found 4AT highly applicable for a remote delirium screening tool over CAM in that the raters require minimal training and K-4AT includes indirect observation and direct cognitive task. These components of K-4AT helps raters evaluate alertness and acute changes even when patients were in a state that direct cognitive tasks cannot be proceeded. Additionally, K-4AT assesses delirious features of patients within the past 24 h, while CAM only evaluates patients’ delirious symptoms at the point of assessment. This has been already recognized as a major weakness of the CAM.

Previous studies have proposed telemedicine for patients with limited access to mental healthcare (i.e., those living in rural areas, attending large schools, or incarcerated in prison), and interactive devices allow diagnosis, tele-psychotherapy, and tele-counseling to be performed by mental health professionals including psychiatrists, psychologists, and physicians (23). The remote delirium evaluation by K-4AT is analogous to telemedicine. In the COVID-19 era, remote assessments and interventions protected both patients and caregivers (24). Recent studies have used remote physical examinations for both the primary care and cognitive assessment of COVID-19 patients (24–26) and a recent mobile platform assesses depression, suicidal ideation, and anxiety (27). However, very few studies have discussed remote screening of neuropsychiatric conditions that are under-recognized such as delirium. Such conditions exert a major influence on the prognosis of COVID-19 and other infectious diseases, and must be tackled early to reduce the risk of complications. Our protocol was written for medical staff but could be modified for use by COVID-19 caregivers, who typically lack medical training. A few studies emphasized that caregivers can detect delirium, and provided guidelines for both general patients (28, 29) and COVID-19 patients (30). Future studies should extend the remote delirium screening protocol to caregivers of COVID-19 patients receiving remote telehealth therapy.

We only verified the inter-rater reliability of COVID-19 delirium diagnoses made by independent medical staff based on the DSM-5 criteria. Although the assessments were not remote, patient contact was limited as the staff were wearing protective clothing and could thus spend little time with each patient. This could have resulted in biased diagnoses. However, the inter-rater reliability of the DSM-5 diagnoses was excellent (κ = 0.710; 95% CI = 0.412–1.000; *p* < 0.001). The K-4AT includes direct measures (i.e., cognitive tasks), re-evaluations by two raters might lead to a practice effect. Moreover, the results from DSM-5-based diagnoses and remote K-4AT evaluations were in agreement, verifying the inter-rater reliability of the K-4AT may not be necessary (31).

This study had some limitations. First, we enrolled general inpatients with various diagnoses in three different settings. The aim was to optimize generalizability, but this may have caused some bias. Small sample size was another limitation of our study, particularly that of the COVID-19 population. Additionally, we had to exclude inpatients’ data for final analysis because they were in a critical condition or ineligible for the study for other reasons, which is a major limitation of most prospective studies. The exclusion of patients with dementia or cognitive impairment may also have created bias. However, to the best of our knowledge, this is the first prospective study to validate the 4AT specifically for remote assessment of delirium in COVID-19 inpatients. Future studies with larger samples should assess delirium both in COVID-19 and uninfected patients with various forms of cognitive impairment. Our remote delirium screening protocol will require further development to that end. Finally, the inclusion of patients with hearing disabilities might lead to false-positives.

In summary, we validated the modified version of the K-4AT, which accurately detected delirium in COVID-19 and general inpatients. Specifically, for COVID-19 patients who require remote evaluation, the K-4AT reliably screened for delirium; the best combination of sensitivity and specificity was obtained at 3, suggesting the optimal cutoff of 3. Finally, we provided a protocol for remote screening of delirium in COVID-19 patients using the K-4AT.

## Data availability statement

The datasets generated and/or analyzed during the current study are not publicly available because the datasets include personally identifiable medical information of all participants, but are available from the corresponding author on reasonable request.

## Ethics statement

The studies involving human participants were reviewed and approved by Institutional Review Board, Seoul National University Bundang Hospital. The patients/participants provided their written informed consent to participate in this study.

## Author contributions

HH and YJ contributed to the analysis and interpretation of data, and drafted this manuscript. HoP, YL, DR, BK, and K-HS contributed to the conceptualization and data curation for this research. HuP contributed to the funding acquisition, conceptualization, data curation, and preparation or the original draft. All authors have read and approved the submission of this manuscript.
